# Endovascular Intervention Among Patients Complicated by Acute Inferior Deep Venous Thrombosis: A Single‐Center Retrospective Cohort Study From Vietnam

**DOI:** 10.1155/ijvm/4819877

**Published:** 2026-03-08

**Authors:** Van Nut Lam, Duc Tin Le, Manh Hung Nguyen, Thuy Vy Tran Thi, Phuc Nhon Nguyen

**Affiliations:** ^1^ Department of Vascular Surgery, Cho Ray Hospital, Ho Chi Minh City, Vietnam, choray.vn; ^2^ Department of Thoracic and Vascular Surgery, Nam Can Tho University, Can Tho, Vietnam, nctu.edu.vn; ^3^ Faculty of Medicine, Hong Bang International University at Ho Chi Minh City, Ho Chi Minh City, Vietnam; ^4^ Cardiothoracic Department, Thong Nhat Hospital, Ho Chi Minh City, Vietnam; ^5^ Department of Internal Medicine, Minh Anh International Hospital, Ho Chi Minh City, Vietnam; ^6^ Tu Du Clinical Research Unit (TD-CRU), Tu Du Hospital, Ho Chi Minh City, Vietnam

**Keywords:** deep vein thrombosis, endovascular intervention, lower limbs, mortality, postthrombotic syndrome

## Abstract

**Background:**

The study was aimed at evaluating the efficacy, safety, and outcomes of endovascular intervention (EVT) among patients with acute inferior deep vein thrombosis (DVT), as well as revealing the risk factors associated with postthrombotic syndrome (PTS) in these patients.

**Methods:**

This retrospective study was conducted at the Department of Vascular Surgery, C.R. Hospital, Vietnam. The study enrolled all the patients diagnosed with acute lower limb DVT and underwent EVT (thromboaspiration, thrombolysis, balloon angioplasty, and stent placement) between January 2017 and December 2022. All the patients were recorded with treatment outcomes, postinterventional complications, and factors relating to PTS on the follow‐up at 1 week and at 1 month after intervention.

**Results:**

A total of 37 patients met the inclusion criteria. Baseline characteristics included a mean age of 55.8 ± 13.3 years, female sex (75.5%), body mass index (BMI) ≥ 23 kg/m^2^ (63.2%), and inferior DVT on the left side (91.9%). Location of thrombus was noted at the iliac vein (97.3%), the femoral vein (70.3%), and the popliteal vein (97.3%). Postoperative length of stay was 5.5 ± 2.7 days, and clinical symptoms reduced in 2.5 ± 0.9 days. There were two cases of bleeding at the interventional site and two cases of death. Partial stenosis at 1 week and 1 month regarding the iliac vein, the femoral vein, and the popliteal vein was 71.4% and 54.3%, 14.3% and 37.1%, and 20.0% and 40.0%, respectively. None of the cases were observed with complete stenosis. In addition, 62.9% of cases reported no PTS. 37.1% of cases were noted with mild PTS. Advanced age, high BMI, duration time of thrombolysis, and underlying diseases were all associated factors relating to PTS. Noticeably, comorbidities and overweight/obesity increased 18.7‐ and 17.33‐fold risk of PTS (*p* < 0.05), respectively.

**Conclusions:**

EVT is an acceptable alternative method in the treatment of acute inferior DVT. A decision‐making of EVTs for acute lower limb DVT should be implemented after assessment of risk factors in large centers with professional conditions and facilities.

**Summary**

• Endovascular intervention is an acceptable alternative method in the treatment of acute inferior deep vein thrombosis (DVT).
• None of the patients were observed with complete stenosis after receiving EVT.
• Advanced age, high body mass index (BMI), duration time of thrombolysis, and underlying diseases were all associated factors developing postthrombotic syndrome (PTS).
• Multidisciplinary teams should be required in the management of acute lower limb DVT.

## 1. Introduction

A DVT is a blood clot that forms within the deep veins. It can occur in the arms and the mesenteric, cerebral, and pulmonary veins [1]. However, the lower extremity deep vein is the most common site [2]. This is a common venous thromboembolic (VTE) disorder with an incidence of 1.6 per 1000 annually. The rate of particular site involvement depends on the anatomical location as follows: distal (40%), popliteal (16%), femoral (20%), common femoral (20%), and iliac veins (4%). The mechanism of DVT could be explained by Virchow triad involving endothelial injury, abnormal blood flow, and hypercoagulability [3]. Major risk factors for thrombosis include older age, surgery, hospitalization, immobility, trauma, pregnancy, the puerperium, hormone use, and endogenous factors such as cancer, obesity, and inherited and acquired disorders of hypercoagulation [4]. Wells criterion could help in the classification of DVT patients [5].

Currently, imaging modalities such as duplex ultrasound, conventional venography, intravascular ultrasound, computed tomography (CT) venography, and MRI play an important role in detecting the location of DVT [6, 7]. Other biochemical markers such as D‐dimer and fibrin monomer and coagulation profiles also contribute to detecting DVT. Thus, it helps in the early management of DVT [8]. Previously, we have had many methods to cure DVT including noninvasive approaches (anticoagulants, thrombolysis) and invasive approaches (percutaneous mechanical thrombectomy, venous angioplasty and stenting, open surgical thrombectomy, and aspiration thrombectomy) [9, 10]. The traditional method of using injectable and oral anticoagulants has been recommended and widely applied. However, this debatable method also shows many limitations, such as the slow recanalization process, leading to prolonged edema and slowing the patient′s recovery process. Moreover, long‐term thrombosis can occur and lead to PTS due to lower limb venous stenosis [11].

To date, endovascular intervention has emerged as a promising method to rapidly remove thrombus from the lower limb venous circulation, thereby helping patients return to normal activity sooner and reducing the risk of developing PTS. This method not only preserves the function of the venous valve leaflets but also allows timely detection and treatment of residual iliac vein stenosis caused by organized thrombus or external compression, such as in May–Thurner syndrome, through the placement of a venous stent [12, 13].

Although endovascular intervention has demonstrated many benefits in the treatment of deep vein thrombosis, there are still difficulties and challenges associated with its implementation, including the safety and efficacy of the treatment. In particular, few studies have focused on identifying factors that influence the outcome of endovascular intervention. This lack of information represents an important gap in clinical practice, as understanding the factors that influence treatment outcomes is fundamental to improving the effectiveness and optimizing patient care. C.R. Hospital is one of the main vascular interventional centers in Vietnam [14]. Our study is aimed at evaluating the treatment results of DVT and its factors affecting the outcome of endovascular treatment of acute lower limb deep vein thrombosis at a single‐center experience.

## 2. Methods

### 2.1. Study Designs

**Figure 1:**
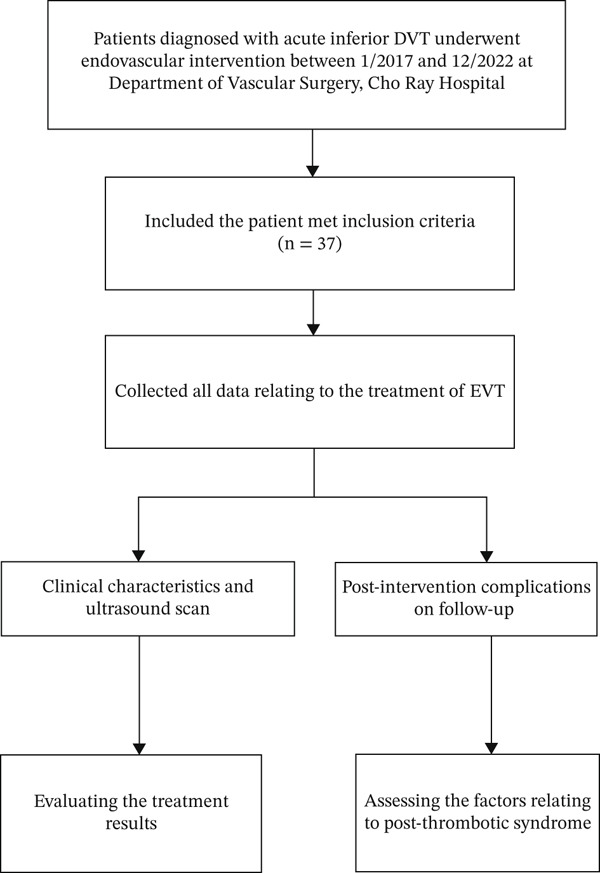
Study flowchart in the present study.

This retrospective cohort study was conducted at the Department of Vascular Surgery, C.R. Hospital, between February and September 2024. The study enrolled all patients diagnosed with acute lower extremity deep vein thrombosis and underwent endovascular treatment from January 2017 to December 2022 (Figure 1). The study was accepted by the ethical council of the institution.

### 2.2. Inclusion Criteria

Patients were diagnosed with lower limb deep vein thrombosis based on clinical manifestations (pain and edema of the lower limb) and confirmed by Doppler ultrasound. The diagnosis was made in the acute stage with the time from clinical onset less than 14 days. Patients were treated with endovascular intervention.

Exclusion criteria included patients with a history of deep vein thrombosis with or without previous treatment, cases with lower limb arterial occlusion, cases with inferior vena cava occlusion, cases with insufficient research data, missing medical file of patient, and all patients who did not return for follow‐up within the first month.

### 2.3. Endovascular Intervention Procedure

All procedures were performed by an experienced specialist in vascular surgery at our department.

1. Patient position: prone position.
2. Machine: C‐Arm OEC 9900 Elite (GE Healthcare) (Figure S1).
3. Anesthesia method: local anesthetize the skin area where the needle is punctured with 2% lidocaine 2 mL.
4. Interventional procedure is as follows:

i. Needle puncture site: usually popliteal vein (or tibial vein).
ii. Under ultrasound guidance, the needle was punctured into the lower left popliteal vein.
iii. Inject 50 IU/kg of heparin intravenously.
iv. Insert the guidewire and 6F sheath into the popliteal vein.
v. Through the guidewire, take a venous image to record the venous occlusion with location, length, and extent.
vi. Proceed to aspirate the thrombus with a syringe through the catheter.
vii. Through the guidewire, insert a soft guidewire (0.035) and a 5F Fountain Infusion catheter (with multiple holes on the side wall) from the common iliac vein to the popliteal vein so that the exit holes are evenly spread and in contact with all the thrombus.
viii. Through the catheter, infuse bolus urokinase solution 4400 IU/kg or alteplase 0.03–0.1 mg/kg.
ix. Continuing to infuse urokinase electric syringe at a dose of 1000–1200 IU/kg/h or alteplase at a dose of 0.01 mg/kg/h (not exceeding 1 mg/h), within 24–72 h, the dose was adjusted according to fibrinogen, hemoglobin, and platelets.
x. Heparin was also infused by electric syringe through the sheath at a low dose of 5–10 IU/kg/h.
xi. Clinically assess the patient′s general condition; monitor symptoms such as fever, pain, and bleeding at the puncture site (if any).
xii. Blood pressure, pulse, and puncture site are checked every 6 h. Hemoglobin and fibrinogen are checked every 12 h, and activated partial thromboplastin time (aPTT) is checked every 6 h to maintain aPTT about 1.5–2 times compared with the control. Heparin was stopped when fibrinogen < 2 mg/dL.
xiii. Doppler ultrasound assessment was done every 1–2 days or angiography after the end of the fibrinolytic treatment.
xiv. Maintain IV heparin infusion for 3–5 days.
xv. In cases where symptoms did not improve, perform angiography and a second intervention after fibrinolytic infusion.
xvi. If a thrombus was detected, a second thrombus aspiration was performed.
xvii. If there was residual stenosis, perform vein dilation with Merit Medical Performa 1050 psi 14/12/10/8 mm balloon and then place a Boston Wallstent 14 × 90 mm if necessary.

### 2.4. Evaluation of Intervention Results

#### 2.4.1. Surgical Complications

1. Local complications: bleeding at the puncture site, swelling, and hematoma.
2. Systemic complications: severe bleeding (brain, digestive tract, …), pulmonary embolism, thrombocytopenia.

#### 2.4.2. Effectiveness of the Intervention

Patients were evaluated at two timepoints:

• At 1 week after discharge by ultrasound. Description of venous lesions was divided into three levels: no stenosis (completely no thrombus), stenosis (thrombus adherent to the wall but still shows blood flow in the vein), and complete obstruction (no blood flow in the vein).
• At 1 month after discharge by clinical assessment according to the Villalta scale and ultrasound scan with the same assessment at 1 week.

### 2.5. Evaluation Criteria

Evaluation of treatment results was at 1 week after discharge. Currently, the method of assessing venous patency is not yet unified worldwide. In our study, venous patency was evaluated according to Enden et al. in the CaVenT study [15]. Venous patency rate was examined by Doppler ultrasound with 5–10 Hz linear probe and/or CT scan with venous contrast. Venous patency was defined as follows:

1. Patency: when the Doppler image is completely clear, or there is an old semimural thrombus and venous reflux, and/or when the CT scan shows images of patency without stenosis or only mild stenosis of the lumen.
2. Obstruction: when the Doppler ultrasound image shows occlusion, the old thrombus completely fills the lumen, no venous reflux is recorded in the main veins, and/or when the CT scan shows images of stenosis or complete occlusion.

PTS is diagnosed when the Villalta score is ≥ 5 points or there is a venous leg ulcer. Grading of PTS was classified as mild: Villalta score from 5 to 9 points; moderate: Villalta score from 10 to 14 points; and severe: Villalta score ≥ 15 points or there is a venous leg ulcer [16].

### 2.6. Data Collection

All the variables were collected on the patient′s medical record. The following information was recorded: age, gender, BMI (kg/m^2^), medical history, clinical symptoms, treatment (intervention method, intervention time), and complications after intervention and during re‐examination. Record clinical signs in retrospective records and during re‐examination according to the Villalta scale. Record paraclinical results: blood test, coagulation, lower limb vascular Doppler ultrasound, and vascular CT.

### 2.7. Statistical Analysis

Variables were classified as continuous and categorical variables. Data was analyzed using Statistical Package for the Social Sciences (SPSS 27) software (IBM Corp., Armonk, New York, United States). Qualitative variables are presented as frequencies and percentages; quantitative variables are presented as mean ± standard deviation or as median and interquartile range depending on normal distribution. Analytical statistics included a chi‐square ( *χ* ^2^ test) or Fisher′s exact test for qualitative variables depending on the number of cases in each cell of a 2 × 2 table and the Wilcoxon signed‐rank test for ordinal variables. A *p* value ≤ 0.05 was considered statistically significant.

## 3. Results

**Figure 2:**
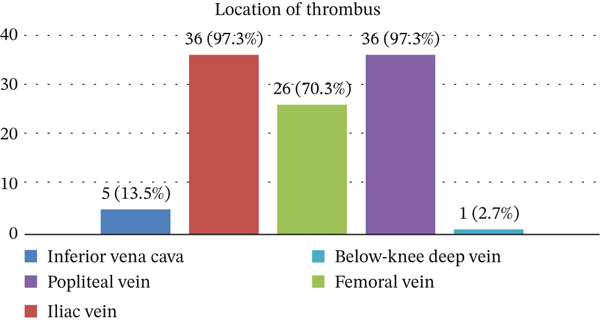
Location of thrombus in the present study.

Among 37 patients, the average age of patients with acute lower limb deep vein thrombosis was 55.8 ± 13.3 years, with the majority of patients in the age group of 40–60 years. Females accounted for the majority with a rate of 75.7%. Nearly half of the cases presented with hypertension. BMI ≥ 23 kg/m^2^ (overweight and obesity) accounted for 63.2%; 91.9% of the cases were related to the inferior DVT on the left side. The common symptoms were pain (86.5%) and swelling (100.0%) (Table 1). The location of thrombus was observed at inferior vena cava (13.5%), iliac vein (97.3%), femoral vein (70.3%), popliteal vein (97.3%), and below‐knee deep vein (2.7%) (Figure 2).

**Table 1:** Baseline characteristics of population in the present study.

Characteristics		Value
Age (year)	Mean ± SD (min–max)	55.8 ± 13.3 (33–80)
	< 20	0 (0.0)
	20–39	5 (13.5)
	40–60	18 (48.7)
	> 60	14 (37.8)

Sex	Male	9 (24.3)
	Female	28 (75.7)

BMI (kg/m^2^)	Normal (18.5–22.9)	14 (37.8)
	Overweight and obese (≥ 23.0)	23 (63.2)

Comorbidities	Hypertension	15 (40.5)
	Diabetes	3 (8.1)
	Ischemic	3 (8.1)
	Others^a^	16 (43.3)

Risk factors	Trauma	4 (10.8)
	Surgery	6 (16.2)
	Contraception	2 (5.4)
	HRT	1 (2.7)

Site of acute inferior deep vein thrombosis	Left	34 (91.9)
	Right	3 (8.1)

Symptoms	Pain	32 (86.5)
	Edema/swelling	37 (100.0)

Onset time of symptoms (days)	Mean ± SD (min–max)	7.7 ± 2.7 (4–13)

Laboratory tests of coagulation disorders	PT (s)	12.2 ± 1.6 (10.1–17.1)
	aPTT (s)	27.7 ± 4.8 (23.0–49.9)
	INR	1.05 ± 0.11 (0.91–1.44)
	Fibrinogen (g/L)	4.29 ± 1.53 (2.03–7.50)

*Note:* Data presented as *n* (*%*) except where otherwise indicated.

Abbreviations: aPTT, activated partial thromboplastin time; BMI, body mass index; HRT, hormonal replacement therapy; INR, international normalized ratio; PT, prothrombin time.

^a^Chronic renal diseases, fibroids, dyslipidemia, lower extremity artery embolism.

Table 2 shows a total of 37 patients undergoing thromboaspiration and thrombolysis as the first intervention. Among them, one case received thromboaspiration, five cases received stent placement, and six cases received balloon angioplasty as the second intervention. Almost all cases were treated with urokinase (94.6%). Due to limited‐resource settings, no patients received inferior vena cava filters (IVCFs) during the study period. Postoperative length of stay was 5.5 ± 2.7 days, range: 2–17 days. Improvement of symptoms was achieved in 2.5 ± 0.9 days, range: 1–6 days. Severe complications were noted with bleeding at the interventional site (*n* = 2) and death (*n* = 2).

**Table 2:** Characteristics of endovascular intervention of lower limb deep vein thrombosis.

Interventions		Value
The first intervention	(Thromboaspiration + thrombolysis)	37 (100.0)

The second intervention	Thromboaspiration	1 (2.7)
	Balloon angioplasty	6 (16.2)
	Stent placement	5 (13.5)

Thrombolysis drug	Urokinase	35 (94.6)
	Alteplase	2 (5.4)

Time use of urokinase (hours)	Mean ± SD (min–max)	50.7 ± 14.3 (24–80)
Dose use of urokinase (UI)		3536.571 ± 1069.947 (1500.000–5940.000)

Duration time of intervention (min)	Mean ± SD (min–max)	117.9 ± 44.2 (60–235)

Site of balloon dilatation	Common iliac vena	6
	External iliac vena	1
	Left femoral vena	3

Stent placement	Left common iliac vena	5

Postinterventional complications	Bleeding at interventional site	1 (2.7)
	Death	2 (5.4)

Length of stay of postoperative day (days)	Mean ± SD (min–max)	5.5 ± 2.7 (2–17)

Improvement of symptoms (days)	Mean ± SD (min–max)	2.5 ± 0.9 (1–6)

*Note:* Data presented as *n* (*%*) except where otherwise indicated. Alteplase: 1 patient used enough dose 100 mg (10 mg bolus, 90 mg infused through catheter), infused over 80 h, and 1 patient died while on therapy: dose 5.84 mg (2 mg bolus, 3.84 mg infused over 4 h). Additional information related to death cases in the study: Patient Vo Thi G. is a case with many high‐risk factors. At the age of 80, with a BMI of 34 (obese) and many serious underlying diseases such as ischemic heart disease, hypertension, and chronic kidney disease, the patient underwent a 200‐min intervention to treat thrombosis in the left popliteal vein. The treatment included the use of urokinase to dissolve fibrin and aspirate the thrombus. However, after the intervention, the patient experienced complications of subcutaneous hemorrhage and decreased hematocrit, leading to the need for a blood transfusion. On the third day after the intervention, the patient′s condition rapidly deteriorated with symptoms of coma, unmeasurable blood pressure, and a sharp decrease in SpO2. Despite intensive resuscitation, the patient died, possibly due to cerebral hemorrhage and severe coagulopathy. Patient Nguyen Thi T., 65 years old, had a series of serious underlying diseases including hypertension, Type II diabetes, chronic kidney disease, and a history of cerebral infarction. The patient underwent an 80‐min intervention to treat thrombosis in the left iliac, femoral, and popliteal veins. After the intervention, the patient was treated with alteplase and heparin to maintain the recanalization process. However, in the evening of the same day, the patient suddenly became comatose and had difficulty breathing, with a decreased SpO2 index and a very high capillary blood sugar index. Consulted with the cardiology department, suspected of pulmonary embolism. The patient was resuscitated and treated intensively and ordered a CT scan of the pulmonary blood vessels and a CT scan of the brain; however, she did not survive.

On follow‐up, none of the patients were observed with complete stenosis of inferior DVT (35/35 cases) on ultrasound scan in 1 week and 1 month. Partial stenosis at 1 week and 1 month regarding the iliac vein, the femoral vein, and the popliteal vein was 71.4% and 54.3%, 14.3% and 37.1%, and 20.0% and 40.0%, respectively. The findings were significantly different between preintervention and postintervention at 1 week as well as postintervention at 1 week and at 1 month (*p* < 0.05) (Table 3).

**Table 3:** Postinterventional result of treatments on follow‐up.

Sites	Timepoints	Ultrasound scan			*p*
		No stenosis *n* (%)	Partial stenosis *n* (%)	Complete stenosis *n* (%)	
Iliac vein	Preintervention	0 (0.0)	0 (0.0)	35 (100.0)	
	1 week	10 (28.6)	25 (71.4)	0 (0.0)	**p** < 0.001^∗^
	1 month	16 (45.7)	19 (54.3)	0 (0.0)	**p** = 0.003^∗∗^

Femoral vein	Preintervention	10 (28.6)	0 (0.0)	25 (71.4)	
	1 week	30 (85.7)	5 (14.3)	0 (0.0)	**p** < 0.001^∗^
	1 month	22 (62.9)	13 (37.1)	0 (0.0)	**p** = 0.011^∗∗^

Popliteal vein	Preintervention	1 (2.9)	0 (0.0)	34 (97.1)	
	1 week	28 (80.0)	7 (20.0)	0 (0.0)	**p** < 0.001^∗^
	1 month	21 (60.0)	14 (40.0)	0 (0.0)	*p* = 0.052^∗∗^

Below‐knee deep vein	Preintervention	34 (97.1)	0 (0.0)	1 (2.9)	
	1 week	0 (0.0)	1 (2.9)	0 (0.0)	*p* = 0.317^∗^
	1 month	0 (0.0)	1 (2.9)	0 (0.0)	*p* = 1.0^∗∗^

*Note:* Data presented as *n* (*%*) except where otherwise indicated. Bold values denote statistical significance at the *p* < 0.05 level.

^∗^Wilcoxon signed‐rank test, between preintervention and at 1‐week postintervention.

^∗∗^Wilcoxon signed‐rank test, between timepoint at 1‐week postintervention and at 1‐month postintervention.

Using Villalta score for assessing PTS, Table 4 shows the mean Villalta score at 4.5 ± 1.3, range: 3–9 points. In general, 62.9% of cases reported no PTS. 37.1% of cases were noted with mild PTS.

**Table 4:** Postthrombotic syndrome after endovascular intervention.

Symptoms	Frequency (*n*)	Percentage (%)
Heaviness		
0	1	2.9
1	32	91.4
2	2	5.7
Pain		
0	6	17.1
1	27	77.1
2	2	5.7
Cramps		
0	17	48.6
1	18	51.4
Pruritus		
0	29	82.9
1	6	17.1
Paresthesia		
0	22	62.9
1	13	37.1
Clinical signs		
Pretibial edema		
0	11	31.4
1	24	68.6
Skin induration	0	0.0
Hyperpigmentation		
0	33	94.3
1	2	5.7
Pain on calf compression		
0	24	68.6
1	11	31.4
Venous ectasia		
0	20	57.4
1	15	42.9
Redness	0	0.0
Venous ulcer	0	0.0
Villalta score	4.5 ± 1.3 (3–9)	
No PTS	22/35	62.9
Mild PTS	13/35	37.1

*Note:* Data presented as *n* (*%*) except where otherwise indicated.

Abbreviation: PTS, postthrombotic syndrome.

In general, clinical factors such as advanced age, high BMI, duration time of thrombolysis, and underlying diseases such as hypertension, diabetes, and renal chronic dysfunction have been noted to influence treatment outcomes and significantly increase the risk of developing PTS. Noticeably, comorbidities and overweight/obese increased 18.7 times and 17.33 times (*p* < 0.05), respectively (Table 5).

**Table 5:** Associated factors in the presence of postthrombotic syndrome.

Factors	*N*	PTS	(%)	OR	95% CI	*p*
*Age*						**p** = 0.037^∗^
20–39	5	1	20	Ref		
40–60	18	4	22.2	1.14	0.10–13.34	*p* = 1.0^∗^
> 60	12	8	66.7	8.0	0.66–97.31	*p* = 0.131^∗^
*Sex*						
Male	9	3	33.3	0.8	0.16–3.94	*p* = 1.0^∗^
Female	26	10	38.5			
*Comorbidities*						
Yes	16	11	68.8	18.7	3.07–113.89	**p** < 0.001^∗∗^
No	19	2	10.5			
*BMI (kg/m^2^)*						
Normal (18.5–22.9)	14	1	7.1	17.33	1.90–157.99	**p** = 0.003^∗∗^
Overweight and obese (≥ 23.0)	21	12	57.1			
*Interventional methods*						
Thrombolysis and thromboaspiration	29	10	34.5	Ref		
Thrombolysis, thromboaspiration, and balloon	1	1	100.0	0	—	*p* = 0.367^∗^
Thrombolysis, thromboaspiration, balloon, and stent	5	2	40	1.27	0.18–8.87	*p* = 1.0^∗^
*Duration time of thrombolysis (hours)*						
≤ 48 h	18	10	55.6	0.171	0.04–0.64	**p** = 0.02^∗∗^
> 48 h	17	3	17.6			
*Onset time (days)*						
≤ 7	16	4	34.6	2.7	0.64–11.47	*p* = 0.172^∗∗^
> 7	19	9	44.4			

Abbreviation: PTS, postthrombotic syndrome.

^∗^Fisher′s exact test.

^∗∗^Chi‐square test.

## 4. Discussion

A total of 37 patients were enrolled in this study. The baseline characteristics in our study were similar to other studies, with a mean age greater than 50 years old; the majority of patients presented with lower extremity pain and swelling. However, sex characteristics remained variable among studies [17]. In the present study, restenosis (reocclusion) was observed mainly in the iliac vein at 1 week (25/35 cases) and at 1 month (19/35 cases). In the femoral vein, the rate of residual venous obstruction was 14.3% (5/35 cases) and 37.1% (13/35 cases) at 1 week and at 1 month, respectively. Other studies found the incidence of stent restenosis was 14.2% during the mean follow‐up time at 36.06 ± 17.66 months [18].

In general, discontinuation of anticoagulants within 1 year (hazard ratio [HR] = 5.03; *p* = 0.048) was the factor associated with acute in‐stent thrombosis [18]. To reduce reocclusion/restenosis, there was routine prophylactic use of antiplatelet drugs: ticlopidine and both low‐molecular‐weight heparin (LMWH) and batroxobin combined with aspirin appearing beneficial at 6 months and at 12 months, respectively. However, the strong evidence remains limited [19].

Of the enrolled patients, there were two cases of death after intervention due to severe hemorrhage and pulmonary embolism. Both patients at ages of 65 and 80 years old had many underlying diseases such as Type II diabetes mellitus, ischemic heart disease, hypertension, and chronic kidney disease. By Society for Interventional Radiology (SIR) criteria, 24% of patients experienced complications (categorized as 10% minor and 14% major). There were also two deaths from intracranial hemorrhage in the study of Karageorgiou et al. [17]. Recently, Qiu et al. demonstrated that residual popliteal vein thrombosis (RPVT) (HR, 4.93; 95% confidence interval, 1.61–15.11) was a significant risk factor for PTS in patients with extensive mixed‐type lower extremity deep vein thrombosis after endovascular therapy [20].

In our retrospective analysis, using the Villalta score, 37.1% of cases were noted with mild PTS. Complications related to PTS were 4.5 ± 1.3 (3–9) points, lower than in the study of Telayna et al. [21]. In the study of Weinburg et al., 692 patients with acute proximal DVT received anticoagulation or anticoagulation plus pharmacomechanical catheter‐directed thrombolysis (PCDT). At 1 month, patients who received PCDT had less residual thrombus compared with control patients, evidenced by noncompressible common femoral vein (CFV) (21% vs. 35%, *p* < 0.0001), the femoral vein (51% vs. 0 70%, *p* < 0.0001), and the popliteal vein (61% vs. 74%, *p* < 0.0001). This study revealed that PCDT results in less residual thrombus but does not reduce venous valvular reflux. CFV noncompressibility at 1 month is associated with more PTS, more severe PTS, and worse QoL at 24 months [22]. In the recent study of Gong et al., clinical success was achieved in 85.2% (75/88) of limbs treated with catheter‐based therapies (CBTs) and 77.5% (31/40) of limbs with conventional catheter‐directed thrombosis (CDT) alone and 88.5% (46/52) in AngioJet rheolytic thrombectomy (ART) and 80.6% (29/36) in large lumen catheter aspiration (LLCA) at the end of conventional CDT. The 12‐month follow‐up showed slightly lower incidences of recurrent thrombosis (7.7% vs. 15.2%) and PTS (14.1% vs. 21.2%), which persisted in patients who underwent ART compared with LLCA (4.3% vs. 12.9% and 8.5% vs. 22.6%) [23].

In the present study, disadvantage factors include advanced age, high BMI, and accompanying disease. Accompanied diseases should be treated carefully in the management of DVT. Noteworthy studies demonstrated that timing and selection of endovascular interventions for DVT remain a crucial issue in the management of DVT and need to be individualized and a multidisciplinary approach in this dynamic field [12, 24]. Multidisciplinary management is an important key to get the right indication of the procedure and an integral patient management [21].

## 5. Strengths and Limitations

Our study was carried out at a tertiary referral hospital with experienced surgeons in the field. The data related to endovascular management remains limited in low‐ and middle‐income countries and low‐resource settings such as Vietnam. Thus, the study helps in sharing the precious experiences and shedding light concerning the clinical outcomes of endovascular treatment in inferior DVT. However, limitations of this study include small sample size, retrospective design, heterogeneous endovascular treatment techniques, lack of advanced techniques such as IVCF, insufficient data to assess long‐term clinical outcomes, lack of independent venographic image review, lack of a control group of patients who did not receive endovascular therapy, and significant losses to follow‐up on the probability of thrombosis‐free survival. In addition, we did not evaluate the quality of life after treatment.

## 6. Conclusion

In summary, endovenous therapy is an acceptable management for acute lower limb DVT. However, multiple factors such as elder age, high BMI, comorbidities, and duration time of thrombolysis should be evaluated carefully to avoid severe outcomes. Further studies with large data and prospective study design were necessary to investigate more long‐term outcomes of endovascular intervention in treating inferior DVT.

**Nomenclature**
BMI: body mass index
DVT: deep vein thrombosis
EVT: endovascular intervention
PTS: postthrombotic syndrome

## Author Contributions

Van Nut Lam, Duc Tin Le, and Manh Hung Nguyen contributed to the conceptualization, methodology, investigation, supervision, administrative procedures, data collection, analysis, and writing of the draft manuscript. Thuy Vy Tran Thi participated in data interpretation and project administration. Phuc Nhon Nguyen was responsible for data analysis, writing, editing, and revising the manuscript. Phuc Nhon Nguyen was the guarantor of this work. Van Nut Lam, Duc Tin Le, and Manh Hung Nguyen contributed equally to this work and share the first coauthorship.

## Funding

No funding was received for this manuscript.

## Disclosure

All authors read and approved the final manuscript. This study does not meet the World Health Organization definition of a clinical trial, so we did not register.

## Ethics Statement

This study was accepted by the Ethical Committee of Ho Chi Minh University of Medicine and Pharmacy with Institutional Review Board Approval Number 244/HĐĐĐ‐ĐHYD on 29th January 2024 and following the declaration of Helsinki.

## Consent

Written informed consent was waived for the retrospective study.

## Conflicts of Interest

The authors declare no conflicts of interest.

## Supporting information


**Supporting Information** Additional supporting information can be found online in the Supporting Information section. Figure S1: Machine C‐Arm OEC 9900 Elite in the present study.

## Data Availability

The datasets used and/or analyzed during the current study are available from the corresponding authors Duc Tin Le and Phuc Nhon Nguyen on reasonable request.
