# Supplementary figures and images for: Endovascular Intervention Among Patients Complicated by Acute Inferior Deep Venous Thrombosis: A Single‐Center Retrospective Cohort Study From Vietnam
Source: Int J Vasc Med. 2026 Mar 8;2026:4819877. doi: 10.1155/ijvm/4819877 (PMC12968076; doi:10.1155/ijvm/4819877)

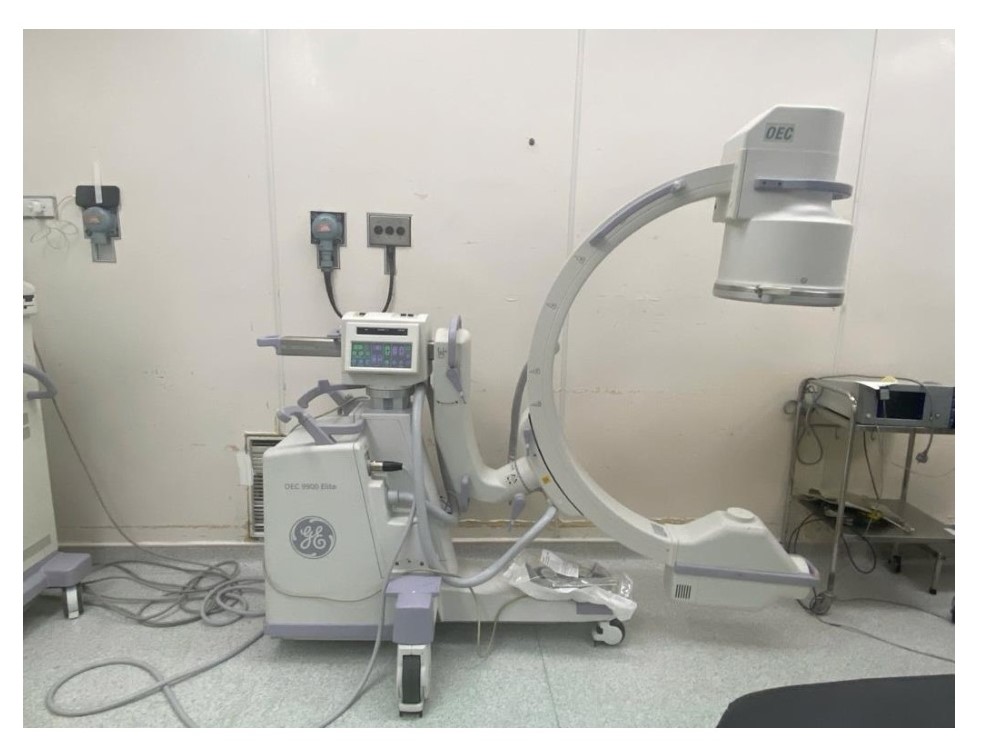

Supplement: Supplementary file 1 — Supporting Information Additional supporting information can be found online in the Supporting Information section. Figure S1: Machine C‐Arm OEC 9900 Elite in the present study. [file IJVM-2026-4819877-s001.jpg]
